# Development and Applications of MOFs Derivative One-Dimensional Nanofibers via Electrospinning: A Mini-Review

**DOI:** 10.3390/nano9091306

**Published:** 2019-09-12

**Authors:** Mingming Liu, Ning Cai, Vincent Chan, Faquan Yu

**Affiliations:** 1 Key Laboratory for Green Chemical Process of Ministry of Education, Hubei Engineering Research Center for Advanced Fine Chemicals, School of Chemical Engineering and Pharmacy, Wuhan Institute of Technology, Wuhan 430073, China; liumingming_cn@outlook.com; 2 Department of Biomedical Engineering, Khalifa University of Science and Technology, Abu Dhabi 127788, UAE

**Keywords:** MOFs, electrospinning, nanofibers

## Abstract

Metal organic frameworks (MOFs) have been exploited for various applications in science and engineering due to the possibility of forming different mesoscopic frameworks and pore structures. To date, further development of MOFs for practical applications in areas such as energy storage and conversion have encountered tremendous challenge owing to the unitary porous structure (almost filled entirely with micropores) and conventional morphology (e.g., sphere, polyhedron, and rod shape). More recently, one-dimensional (1D) MOFs/nanofibers composites emerged as a new molecular system with highly engineered novel structures for tailored applications. In this mini-review, the recent progress in the development of MOFs-based 1D nanofibers via electrospinning will be elaborated. In particular, the promising applications and underlying molecular mechanism of electrospun MOF-derived carbon nanofibers are primarily focused and analyzed here. This review is instrumental in providing certain guiding principles for the preparation and structural analysis of MOFs/electrospun nanofibers (M-NFs) composites and electrospun MOF-derived nanomaterials.

## 1. Introduction

MOFs are synthesized by the incorporation of both inorganic and organic units through template-guided assembly (e.g., mesh synthesis). The design flexibility of MOF in terms of highly variable geometry and functionality based on the choices of incorporated constituents has attracted increasing attentions from researchers in catalysis, energy, and electronics. The main advantage of MOFs in comparison with conventional porous materials is the achievement of specific structure and tunable porosity at the molecular level by adjusting both metal species and organic ligands. For instance, the typical porosity of MOFs is higher than 50% of the total solid volume while the surface area of MOFs generally ranges from 1000 to 10,000 m^2^ g^−1^, both superior to those found in conventional porous materials such as carbons and molecular sieve [1]. 

Until now, most studies in MOF have been steered towards the development of potential applications in important technological areas, such as catalysts [2,3,4], carbon dioxide capture [5], gas storage/separation [6,7,8,9], sensors [10,11], etc. However, many challenges remain to be resolved for advancing various emerging applications of MOF based devices. For instance, crystalline MOF is fragile and easy to be disintegrated into fine powder, which causes a series of problems in adsorption and separation process, such as pipeline blockage, separation efficiency, low MOF recovery [12]. In catalytic process, metal nanoparticles within the MOF matrix tend to agglomerate due to the large surface tension, resulting in a significant reduction in catalyst activity. Moreover, the structural stability and conductivity of MOF materials are difficult to meet the stringent requirements of battery materials.

In order to expand the applications of MOFs, more attention has been paid to the design and fabrication of 1D MOFs/nanofibers composites and their derivatives [13]. For example, MOFs/nanofibers can be carbonized to produce MOFs-derived carbon nanofibers. This new class of MOFs/nanofibers and their carbonized products contain abundant active sites, which are advantageous to the improvement of reaction activity. At the same time, the coexistence of mickle micropores and mesopores significantly enhances the adsorption capacity and electrical conductivity during catalytic process. In addition, the stability of MOF derivative nanofibers composites is enhanced, which is attributed to the protective effect of nanofiber component and the increased cohesiveness between nanofibers and active components [14].

While various methods have been introduced for the synthesis of MOFs/nanofibers composites and their derivatives, it is worthwhile to mention that electrospinning has emerged as the most reliable technique for preparing MOFs/electrospun nanofibers (M-NFs). In general, electrospinning applies a strong electric field upon a polymer solution or polymer melt under active dispensation to nanofibers of 2 nm to several micrometers in diameter onto designated substrates [15,16]. Electrospun nanofibers are characterized by three-dimensional (3D) porous framework and nano-structure, which are crucial properties for providing unique capabilities in various applications [17,18]. Thus electrospinning technique enables the preparation of new materials with multifunctional properties including 3D architecture, nanoscale structure, and chemical functionality, which are arduous to achieve in other alternative processes—e.g., thermally-induced phase separation [19]. More importantly, electrospinning opens the possibility to prepare a variety of classes of novel materials—such as polymer alloys, nanoparticles, active agents, etc. In recent years, there have been several reviews focusing on MOFs [20,21] as well as electrospinning [22,23,24,25,26]. To date, there were few reviews on M-NFs and their carbonized products (MOFs-derived carbon nanofibers, M-CNFs). A recent review published in *Materials Horizons* solely discussed the applications of MOF-derived porous or hollow carbon-based nanofibers for energy storage and conversion [13]. Only a limited information about MOF/nanofibers was provided in the above review [13]. Due to their attractive properties for respective applications in different fields, a systematic survey and analysis of the synthesis and applications of electrospun MOF nanofiber materials and electrospun MOFs-derived carbon nanofibers is required, which is instrumental for researchers in multidisciplinary fields ranging from material sciences and catalysis to electronics.

## 2. MOFs-Derived Nanofibers via Electrospinning

### 2.1. ZIFs/Electrospun Nanofibers (Z-NFs)

Zeolitic imidazolate frameworks (ZIFs) are a typical class of MOFs characterized by its porous crystalline structure extending into 3D space by bridging each tetrahedral metal ion with multiple imidazolate (Im) groups. Due to the flexibility of metal ions and organic blocks, the structure and properties of zeolites can be forged with interesting properties for challenging applications in molecular catalysis and separation [27]. As such, ZIFs have become the preferred material for preparing M-NFs based on relatively its simple synthesis, design flexibility, and structural specificity. The preparations and applications of M-NFs have been extensively studied in recent years [14,28,29,30,31,32,33,34]. At present, there are two main methods for preparing M-NFs: 

(I) In situ growth method by preparing electrospun nanofibers that are incorporated with contain metal ions (or ligands), followed by in situ growth of MOF nanoparticles on electrospun nanofibers; 

(II) Co-electrospinning synthesis by mixing MOF nanoparticles with a polymer solution to form a precursor followed by formation of M-NFs via electrospinning. 

**Figure 1:**
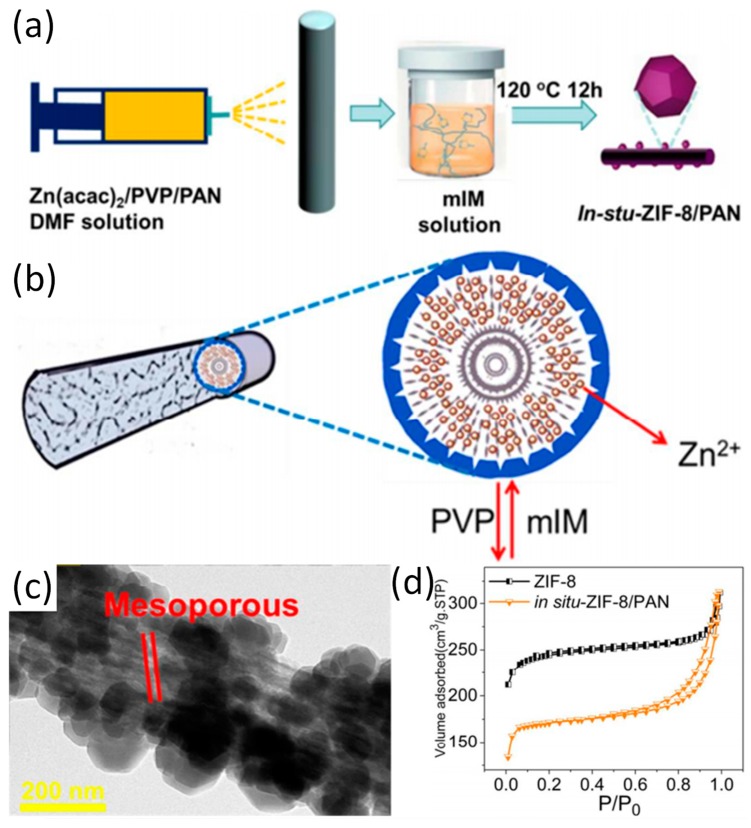
(**a**) The schematic diagram of in situ growth of ZIF-8 on PAN; (**b**) The formation mechanism of in situ ZIF-8/PAN fibers; (**c**) TEM patterns of the in situ-ZIF-8/PAN; (**d**) The N_2_ adsorption and desorption measurements of in situ-ZIF-8/PAN and ZIF-8. Reproduced from [14], with permission from American Chemical Society, 2018.

First of all, the preparation of zeolitic imidazolate framework-8 (ZIF-8)/polyacrylonitrile (PAN) nanofibers is introduced as a model M-NFs by in situ growth method (Ⅰ) was highlighted [14]. Specifically, as depicted in Figure 1a, zinc acetylacetonate (Zn(acac)_2_), PAN, and poly-(vinylpyrrolidone) (PVP) are mixed in *N,N*-Dimethylformamide (DMF) to obtain an electrospun precursor solution. Then Zn(acac)_2_/PVP/PAN fibers are prepared by electrospinning immediately followed by immersion in mixed with 2-methylimidazole (mIM) solution within a Teflon-lined container and kept at 120 °C for 12 hours. Because PVP is more hydrophilic than PAN, PVP can be extracted from Zn(acac)_2_/PVP/PAN fibers in accompany by the generation of pore structure by hydrothermal treatment (Figure 1b). It was shown that ZIF-8 nanocrystals grow tightly on the surface of PAN nanofibers. The mesoporous structure of in situ-ZIF-8/PAN is displayed on the surface of nanofibers during in situ growth process, which may be attributed to the removal of PVP (Figure 1c). Interestingly, a typical behavior of type IV hysteresis loop as shown in Figure 1e validates the existence of mesopores on ZIF-8/PAN fibers. Recently, mIM/PAN nanofibers have been prepared by electrospinning and subsequent chelating with Zn^2+^ ions provided by zinc acetate, leading to the formation of core–shell PAN@ZIF-8 [35]. Addition of mIM and zinc acetate solution during in situ growth can promote the further growth of ZIF-8 nanocrystals. Moreover, the core–shell structure of the composite displays high toughness due to the support of underlying PAN nanofibers. With regard to the in situ growth method in obtaining Z-NFs, organic ligand/polymer NFs or metal ion/polymer NFs are prepared first, and then the ZIFs crystal grows on the respective NFs.

Apart from in situ-grown ZIF-8/PAN, other types of Z-NFs prepared have been prepared by in situ growth method. Zhou and co-workers reported the first time in situ crystal growth of ZIF-8 on electrospun polyurethane (PU) nanofibers (ZIF-8/PU) [34]. More specifically, the surface of PU nanofibers were roughened by the treatment with concentrated sulfuric acid and chromic anhydride, exposing more binding sites on the nanofiber surface. For instance, the emergence of anionic groups on the surface of PU nanofiber facilitates the adsorption of Zn^2+^, which serves as the coordination center of the ZIF-8 nanocrystal to be eventually distributed in high concentration on the PU nanofibers. Moreover, BET surface area of ZIF-8/PU decreases with the amount of ZIF-8 nanocrystal. Although the loading of the MOFs nanoparticles was increased by activating the surface of the fiber and the like, which brought about an occlusion of the pores and attenuate the adverse effects of the one-dimensional morphology of the nanofibers. Therefore, it is not advisable to increase the load blindly, various factors such as the ‘bearing capacity of the nanofibers’ should be comprehensively considered in the preparation of M-NFs. 

In addition to pre-treating the materials during in-situ growth to optimize the structure of the M-NFs, different metal sources can also be used to obtain different structures and performances. Different types of materials can be selected as the source of metal ions or as templates for the growth of ZIFs crystal. By introducing other materials into the crystal growth process, a variety of composites with unique structures and physical properties can be obtained. Activation of the fiber surface and continued addition of ligand or metal ion solution during in situ growth can promote the ZIFs crystal growth. When In_2_O_3_/ZnO_2_ nanofibers instead of Zn^2+^ coated PU nanofiber was applied as a source of Zn^2+^ for serving as a template for the in situ growth of ZIF-8, an In_2_O_3_/ZIF-8 core–shell complex was successfully produced with the effective loading of ZIF-8. It is also noteworthy that the transformation from ZnO particles to metal centers coordinated with organic ligands may likely lead to the formation of structural defects between linkers, thus enlarging the pore size of metal–organic framework ZIF-8 [36,37]. In addition, the transformation as mentioned above can cause ZIF-8 to form multiple pore structures. 

**Figure 2:**
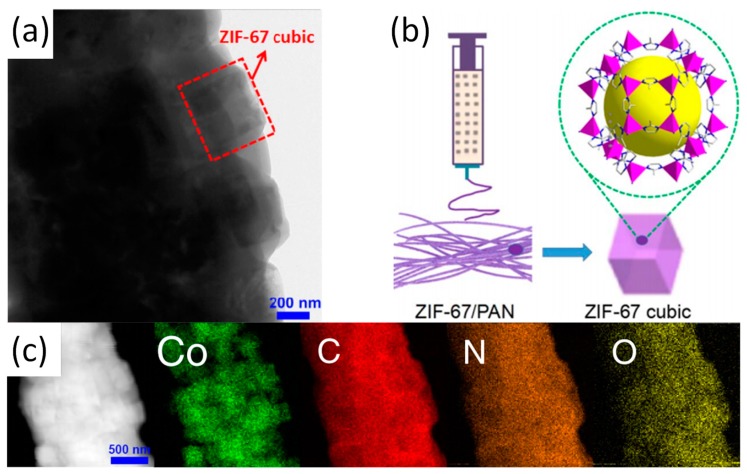
(**a**) TEM image of ZIF-67/PAN; (**b**) Preparation schematic of ZIF-67/PAN; (**c**) STEM image and the corresponding elemental mapping of ZIF-67/PAN. Reproduced from [38], with permission from Elsevier, 2017.

While the in situ growth method is being developed, another method known co-electrospinning has received increasing attentions. Again, ZIF-67/PAN is used as a typical example to cover the co-electrospinning method in detail [38]. Schematic diagram, TEM image and the corresponding elemental mapping of ZIF-67/PAN as shown in Figure 2. After the synthesis of ZIF-67 nanoparticles, the nanoparticles were mixed with PAN to form an electrospun precursor solution and ZIF-67/PAN nanofiber was fabricated by electrospinning. When the ZIF-67/PAN are applied to the catalytic degradation of pollutants, the high specific surface area of the ZIF/PAN can promote effective contact between active sites and pollutants. Random arrays and macroscopic materials with continuous specific surface area and flexibility are also promotive to the recovery and reuse of catalysts [39]. In addition to the most common only used PAN and PVP, the use of other polymers such as polyethylene oxide (PEO) and polystyrene (PS) has improved the fiber stability and expanded the range of possible applications. Previously, Smarsly and co-workers have reported on ZIF-8/PVP nanofibers, which was prepared by co-electrospinning [33]. Table 1 lists the BET surface area of the nanofibers formed by the combination of ZIF-8 and different types of polymer. The co-electrospinning method enables ZIFs nanoparticles to be uniformly loaded on electrospun fibers. It is worth noting that the co-electrospinning method imposes design constraints on the selection of ZIFs within a specific range of size. If ZIFs is too large compared to the electrospun fibers with dimension of several hundred nanometers, it would result in the ineffective encapsulation of ZIF by the electrospun fiber matrix and nonuniform dispersion of the particles on the fiber matrix. Comparatively, the ZIFs particles with appropriate size would be evenly dispersed in the electrospun solution before electrospinning to ensure uniform loading of the particles on electrospun fibers. Regardless of the method route of synthesis of the Z-NFs, it has the following characteristics in general:

(a) ZIFs nanoparticles are evenly loaded in the fiber matrix and the degree of loading is adjustable, which effectively avoid the large-scale agglomeration of ZIFs nanoparticles;
(b) Defects, mesopores, etc. can be formed during electrospinning;
(c) Nanofibers can enhance the mechanical properties and stability of Z-NFs due to its support and protection of ZIFs nanoparticles.

### 2.2. Other Types of M-NFs 

Z-NFs is not only the most representative type of material in M-NFs, but also the earliest and most widely studied materials. With the development of Z-NFs, more MOF materials other than sole ZIFs have been produced through the fabrication of nanofiber composites by electrospinning (in the rest of the article, M-NFs only refers to other types of M-NFs except Z-NFs). M-NFs are prepared in a similar way compared to Z-NFs, while larger range of structural and physical properties of M-NFs are revealed, due to the significant variability among different metal centers and organic ligands [40,41,42,43,44,45,46,47]. 

**Figure 3:**
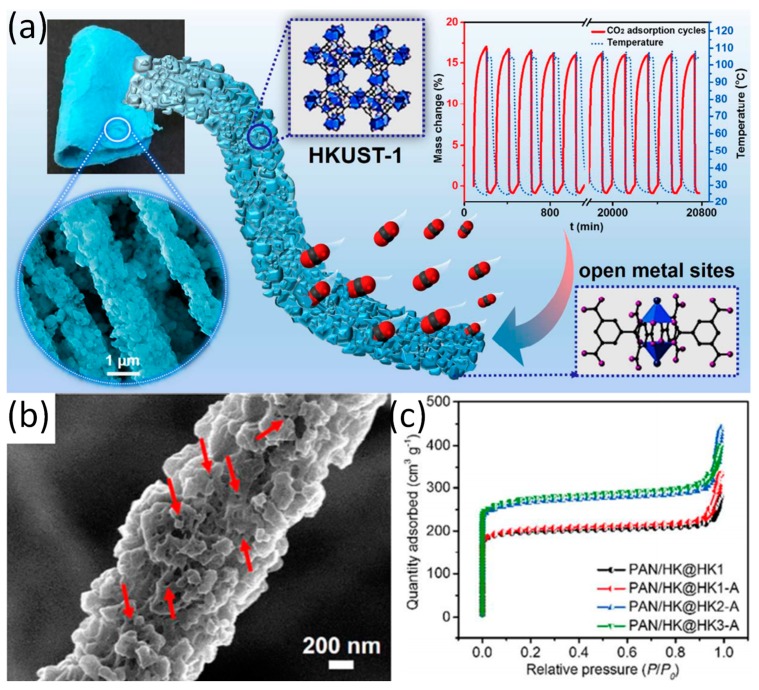
(**a**) Overview of HKUST-1/PAN and adsorption of CO_2_; (**b**) SEM image with higher magnification of the HKUST-1/PAN nanofiber; (**c**) N_2_ adsorption–desorption isotherms of samples with different growth times. Reproduced form [40], with permission from American Chemical Society, 2017.

The preparation methods of ZIFs are simple and the preparation conditions are mild. However, the preparation conditions of MOFs such as HKUST-1 are complicated. Thus, the preparation of such M-NFs has a variety of methods. For instance, Wang and co-workers have prepared a new type of M-NFs known as HKUST-1 (Cu-MOF)/PAN nanofibers by combining co-electrospinning and in situ growth, which promoted multiple growth and activation processes [40]. The active site of CO_2_ adsorption on the HKUST-1/PAN nanofibers are mainly at the open metal sites (Figure 3a). The as-synthesized HKUST-1 particles and HKUST-1/PAN NFs readily exhibits polydisperse microporous characteristics (Figure 3b). As shown in Figure 3c, the BET surface area of the HKUST-1/PAN NFs increase significantly due to the increase of the loading of HKUST-1 nanoparticles after three times of growth and activation. Interestingly, the multiple growth and activation processes during the preparation of HKUST-1/PAN nanofibers with high HKUST-1 can promote the effective integration between coordinated unsaturated metal sites and uncoordinated –COO^-^ within frameworks [48]. In addition, the membranous HKUST-1/PAN nanofibers solves the problem of pipeline blocking and recovery inefficiency during the adsorption process as the nanofiber is less susceptible to powder and display less tendency to form agglomerate [49,50]. 

Multiple activations and growths can enhance the loading of MOF nanoparticles and the binding force of MOF particles and nanofibers to obtain more stable and more active composites. Besides, similar performance can be achieved by selecting the appropriate polymer with a target group which can form a strong bonding with the organic functional groups or ions in MOFs. Recently, Peng and co-workers have reported a UiO-66/PVA (polyvinyl alcohol) nanofiber networks via co-electrospinning [41]. The hydroxyl groups (-OH) in PVA and the carboxylic acid groups (-COOH) in UiO-66 could be esterified during the electrospinning process to form ester bonds, which facilitates the formation of interwoven nanofiber networks. Moreover, the nanofiber networks can improve the wettability of materials in the electrolytes when it is applied as battery materials [51]. It is tough to fabricate independent MOF–organic matrix composites with high MOF-loading capacity by general preparation method. Recently, Wu et al. have reported the fabrications of HKUST-1/PS (polystyrene) and MIL-101(Fe)/PS via co-electrospinning when most MOF particles are encapsulated within the fibers during electrospinning [42]. Only when MOF particles with high loading enough are exposed to the surface of the fibers, the carrying of gas and the transfer of electron can be facilitated for the materials. However, adding high load of MOF particles will reduce the suitability of polymer solutions for use in electrospinning. In addition, with the increase of MOF loading during electrospinning, it is difficult to obtain compact and continuous MOF films. By using MOF-doped electrospun nanofibers as the skeletal backbone, the growth of second MOF layer on the fibers can overcome the existing difficulties [42]. In short, the problems of preparing M-NFs can be resolved by a combination of co-electrospinning and in situ growth. 

The polymers related to M-NFs which were reported in recent years are mainly PAN, PVP, PVA, PS, etc. However, many other polymers (such as PVDF, PA, PEO, etc.) are also important materials for preparing M-NFs. Laurila et al. reported the in situ crystal growth of HKUST-1 on electrospun cellulose nanofibers [52]. Yang et al. also reported on a composite material, which was composed of cellulose paper and MOF-5 (Zn_3_(BDC)_2_) metal organic frameworks (paper@MOF-5) materials [53]. Zhao et al. reported that TiO_2_ coatings deposited on polyamide-6 (PA-6) nanofibers by atomic layer deposition (ALD) to form Zr-based MOF thin films (PA-6@TiO_2_@UiO-66-NH_2_). Moreover, certain MOF–nanofiber textile composites can achieve ultra-fast degradation of chemical warfare agents (CWAs) [54]. Wu et al. reported that electrospun nanofiber membrane composed of MOFs and SPPESK was applied in proton exchange membrane fuel cell [55]. Recently, Xu et al. reported on the application of ZIF-8 functionalized hierarchical micronanofiber membrane (PVDF-g-ZIF-8) in high-efficiency oil/water separation [29]. Rosal and co-workers presented polylactic acid (PLA) fibers containing cobalt-based MOF as a new class of antimicrobial film material [31]. Similarly, Shariatinia and co-workers reported on the synthesis of antimicrobial chitosan–polyethylene oxide (CS-PEO) nanofiber mats loaded with ZIF-8 NPs via electrospinning [56]. Table 2 lists the application performances or properties of different types of MOF/electrospun nanofibers in recent years.

## 3. Electrospun MOFs-Derived Carbon Nanofibers

### 3.1. Synthesis

Electrospun MOFs-derived carbon nanofibers (M-CNFs) are generally prepared by heat treatment and reduction of M-NFs that obtained via electrospinning. After M-NFs are obtained, the heat treatment of composites can be roughly classified into two categories: 

(I) The composites are directly subjected to high temperature carbonization under an air atmosphere to obtain a target product (direct calcination method). The structure and properties of the target product can be controlled by changing the temperature, heating rate, and calcination time. 

(II) Preheating the composites in an air atmosphere at lower temperature and then transferring the composites to high temperature carbonization under N_2_ (or Ar) atmosphere (indirect calcination method).

In the direct calcination method, pure phase metal oxides can be obtained at higher temperatures. Recently, Kim and co-workers have reported that the as-spun Pd@ZIF-8/PVP/Sn composite NFs were transformed to PdO@ZnO-SnO_2_ NTs after the calcination at 600 °C for 1 h in air atmosphere under the heating rate of 10 °C min^−1^ [61]. Some polymer fibers (such as PAN) are easily decomposed at high temperatures, causing the damage to the fiber structure. Therefore, in the indirect calcination method, before the calcination of samples at high temperature, the samples are preheated and oxidized first to remove some heat-labile organic functional groups to ensure the integrity of the nanofiber network structure. For instance, Guo et al. reported the obtention of the Co/CoO_x_-N-C via the pyrolysis process that includes stabilization and carbonization. The progress of stabilization was implement in air at 280 °C for 2 h. Carbonization step was conducted in a N_2_ atmosphere at temperature up to 800 °C for 1 h [62].

### 3.2. Application

The wide application of M-CNFs is attributed to its attractive structures such as one-dimensional nanofibrous morphology and hollow tubular structure. MOFs-derived carbon nanofibers have been considered as excellent electrode materials in energy conversion and storage devices [63,64,65]. Moreover, catalysis and sensors are also the main applications of M-CNFs. Typical applications (e.g., battery, sensor, and electrocatalyst) of materials are presented and analyzed.

#### 3.2.1. Battery

• Lithium/sodium ion battery

The difference in physical properties of electrode material directly affects the performance of the battery. Poor battery performance is mainly attributed to two aspects: (1) Its extremely low intrinsic electrical conductivity; (2) Volume expansion of the material during charging/discharging, which causes particle comminution and spalling [66]. Many studies have focused on the development of electrode materials with high specific capacity, good rate performance and stability. For instance, Yi et al. reported on a Mn_3_O_4_/graphene composites as electrode of battery [67]. Although the material has high reversible capacity and current density, due to the high surface energy and van der Waals forces, irreversible overlap and condensation of graphene nanosheets easily occur during charging and discharging. Wang et al. reported that preparation of carbon-coated manganese oxide (Mn_3_O_4_@C) via solvothermal reaction of manganese acetate monohydrate and polyvinylpyrrolidone [68]. However, the active material is encapsulated in carbon during solvothermal process, which will result in a reduced contact interface between the active material and the electrolyte. M-CNFs as a new class of carbon materials have received more and more attention due to their outstanding performance, and the supporting effect of the carbon nanofiber networks on the system can improve the resistance of the composite towards volume change. Moreover, the carbon nanofiber networks can uniformly load the MOF nanoparticles, which increase the contact area between the composite and the electrolyte. For instance, Yang et al. a porous Co_3_O_4_ with electrospun carbon (Co_3_O_4_/C) prepared by annealing the ZIF-67/PAN templates, the Co_3_O_4_/C shows a capacity of 1024.1 mAh g ^−1^ after 100 cycles [69]. 

**Figure 4:**
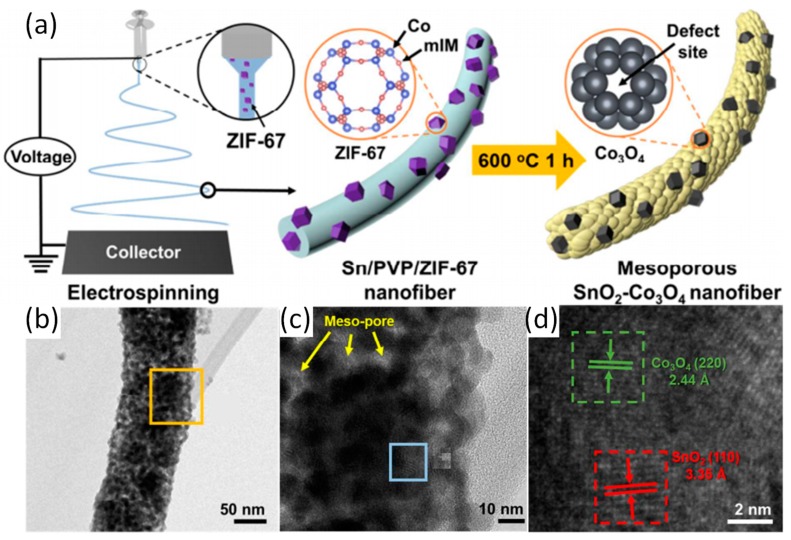
(a) Schematic illustration of synthesis process of SnO_2_-Co_3_O_4_ NFs; (b) TEM image of SnO_2_-Co_3_O_4_ NFs; (c) and (d) HRTEM image of SnO_2_-Co_3_O_4_ NFs Reproduced form [70], with permission from American Chemical Society, 2018.

**Figure 5:**
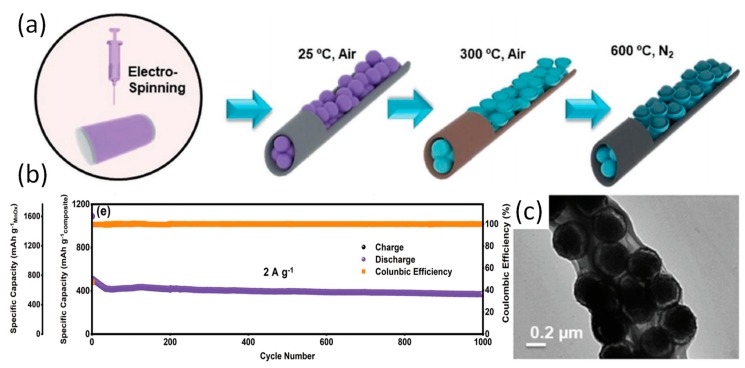
(**a**) Schematic fabrication process of ysMnOx@NC; (**b**) Prolonged cycle stability of samples at the current density of 2 A g^−1^; (**c**) TEM images of ysMnOx@NC. Reproduced from [72], with permission from Wiley, 2019.

Electrospun MOFs-derived one-dimensional carbon nanofibers are ideal materials for battery electrode materials due to their high specific surface area, good electrical conductivity, and well-defined porous structure. For instance, Cheong et al. have prepared one dimension SnO_2_-Co_3_O_4_ NFs with ZIF-67 (which contains Co ions and mIM) as a template [70]. Schematic illustration of synthesis of SnO_2_-Co_3_O_4_ NFs are shown in Figure 4a. The one-dimensional SnO_2_-Co_3_O_4_ NFs was formed via electrospinning and subsequent calcination process form a large number of mesoporous structures. The results shows that the introduction of the ZIF-67 framework not only causes the material to form a large number of oxygen vacancies, but also enhances the conductivity of the material, inducing more active sites for Li storage in lithium-ion batteries (LIBs). As such SnO_2_-Co_3_O_4_ NFs maintain a reversible capacity of 1287 mAh g^−1^ after 300 cycles at a current density of 500 mA g^–1^_._ Similarly, the authors have prepared hollow porous SnO_2_-CuO composite nanotubes by electrospinning and calcination [71]. After the introduction of the MOF framework as a template and co-electrospinning with polymers, the formed SnO_2_-M_x_O_y_ (metal oxide) nanocomposites (nanofibers) display a porous (mesoporous) structure as well as a large number of defects and oxygen vacancies, which solves the problems of poor cycle stability, low ionic conductivity, and low reversible capacity of SnO_2_. In addition to the introduction of MOF framework into SnO_2_ system by electrospinning and heat treatment to form SnO_2_-M_x_O_y_ porous nanofibers, many reports have focused on the formation of metal oxide/carbon nanofibers composites formed by co-electrospinning of MOF nanoparticles and polymer and the subsequent heat treatment as electrode materials for batteries. Yang et al. have reported a yolk–shell MnOx carbon nanofiber composite (ysMnOx@NC) based on Mn-MOF [72]. The yolk–shell structure of the material can be seen from the Figure 5d. The structure can accommodate volume changes during material cycling and reduce loss of activity. It is shown that ysMnOx@NC retains great activity even after 1000 cycles (current density of 2 A g^−1^). Double buffering of nanofibers and the yolk–shell structure can reduce the activity due to material loss during cycling, thereby increasing the reversible capacity of the battery.

Preparing materials with special structure (porous or yolk shell structures) is a way to improve the performance of M-CNFs as battery materials. Besides, preparation of bimetallic or polymetallic composite materials is also an important method to optimize the performance of M-CNFs. The improvement in performance is mainly due to the synergy between bimetallic or polymetallic. Sodium-ion batteries (SIBs) are also of much interests. Recently, Li and co-workers have reported a kind of hierarchical heteroatom-doped carbon nanofibers decorated with several nanocomposites (NiCo_2_O_4_/NiO/carbon nanofibers) based on Co/Ni-ZIF, which was prepared via successive carbonization and oxidation heat-treatments [73]. One-dimensional carbon nanofibers are intertwined by the MOF framework to form a 3D conductive matrix, and uniform dispersion of the active material therein can also improve electrical conductivity and cycle stability. The material showed a high sodium storage capacity of up to 210 mAh g^−1^ after 200 cycles (100 mA g^−1^). Zhang et al. have also prepared carbon fibers with uniformly distributed Co_3_O_4_ hollow nanoparticles (NPs) by electrospinning of ZIF-67 NPs and thermal treatment [74]. 

To sum up, typical composite materials are generally obtained by using the MOF as a template via electrospinning and the subsequent heat treatment or reduction process. When the material is applied to the LIBs and SIBs, the performance is improved mainly for the following reasons: 

(a) The MOF framework will form excess oxygen vacancies and defects, thereby increasing the active site of the material during post-treatments; 

(b) The porous interwoven network of MOF derivative carbon nanofiber can improve the mass/charge transport and the conductivity of the material, support the composite system, and adapt the cyclic change of the material to reduce the loss of activity of the material for achieving high lithium (sodium) storage capacity;

(c) Synergistic interaction between polymetallic and between metal and carbon nanofiber networks can improves material performance;

(d) Anisotropic materials with hollow and porous structures have large specific surface area and strong carrying capacity.

• Metal-air battery

Zn-air battery is the most widely studied and used battery in the metal-air battery category. MOFs are considered as a suitable material for Zn-air battery due to its tunable structure and inherent heteroatoms that promote electron transfer. However, MOF-derived carbon catalysts are generally susceptible to evolution into isolated nanoparticles, which greatly reduces conductivity and thus weakens its electrochemical properties. One-dimensional porous conductive networks of carbon nanotubes or carbon fibers have been shown to improve the conductivity and electron transfer of materials, which has aroused widespread interest among researchers. It is worth mentioning that composite materials like M-CNFs with hierarchical structure and hybrid nanostructures provide additional channels for material diffusion, promoting exposure of active reactive sites [75]. 

#### 3.2.2. Supercapacitor

According to different energy storage mechanisms (SCs), supercapacitors can be divided into two categories: electric double layer capacitors (EDLCs) and Faraday quasi capacitors. EDLCs has received much attention for its outstanding advantages [76]. Carbon materials with a porous structure and a large surface area are necessary to improve the performance of EDLCs. Among the conventional carbon materials—including carbon nanotubes [77], graphene [78,79], and carbon nanofibers (CNFs) [80,81]—many of them have been widely used as electrodes for EDLCs. Kim et al. reported a carbon materials with a high specific surface (1453 m^2^ g^−1^) by activated carbon fiber prepared [82] The introduction of nitrogen not only increases the energy density of the composite but also promotes the conduction of electrons [82,83]. M-CNFs are well suited for application as supercapacitor electrode materials because of their large specific surface area with abundant pore structure and inherent N-doping. For instance, Yao et al. reported the nitrogen-enriched hierarchical porous carbon nanofibers (NHCFs) prepared by electrospinning the composite of ZIF-8 and PAN, and at a current density of 40 A g^−1^, the specific capacitance was maintained at 146.7 F g^−1^ [84]. Recently, the author also reported on the N-doped graphitic hierarchically porous carbon nanofibers which were obtained by electrospinning a composite of bimetallic organic framework (Zn/Co-MOF) and polyacrylonitrile. The Zn/Co-MOF-based carbon nanofibers exhibited a high capacitance of 326 F g^−1^ at 0.5 A g^−1^ [85].

#### 3.2.3. Sensor

**Figure 6:**
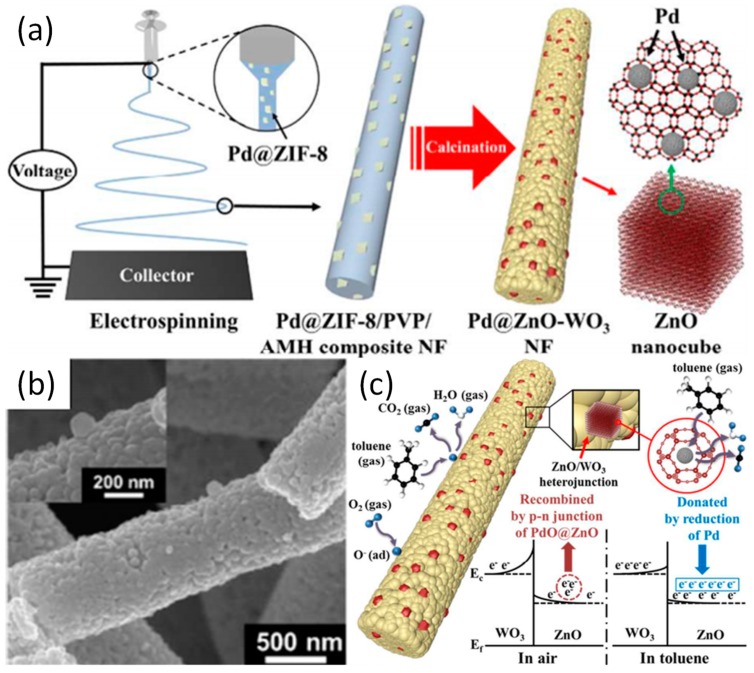
(**a**) Preparative process diagram of the Pd@ZnO-WO_3_ NFs; (**b**) TEM image of Pd@ZnO-WO_3_ NFs and high magnification TEM image; (**c**) Schematic illustration of sensing mechanism for Pd@ZnO-WO_3_ NFs. Reproduced from [90], with permission from American Chemical Society, 2016.

Nowadays, air pollution is getting more serious, the monitoring of volatile organic pollutants such as acetone is receiving more attentions. MOFs have many attractive features, including the ability to encapsulate precious metal nanoparticles (metal@MOF) [86]. Hermes et al. have reported that metal organic chemical vapor deposition was used to release metal atoms from precursor and penetrate metal atoms into MOFs [87]. Jiang et al. synthesized the NPs@ZIF-8 coated with nanoparticles via sequential deposition and reduction of metal precursors [88]. However, the application of these methods is limited by the relatively poor chemical stability of MOF, which is mainly attributed to thermal instability of organic ligand in MOFs. It is worth noting that the heterojunction formed between the carbon nanofibers and the active material from MOF, which can improve the stability of the composite. Moreover, the reduced clustering of precious metal nanoparticles in composite nanofiber matrix can improve the sensing performance of the material. In addition, electrospinning functionalizes catalysts onto one-dimensional nanofibers to provide high specific surface area and high gas accessibility as required for high performance chemical sensors [89]. For instance, Koo et al. have reported on the heterogeneous sensitization of metal-embedded metal oxide (M@MO) composite catalysts driven by MOF on semiconductor metal oxide (SMO) nanofibers (NFs) via electrospinning and calcination [90]. During calcination, metals are oxidized to a metal oxide while the framework of the MOFs remains and the nanoparticles are fixed onto the framework. Electrospinning enables the immobilization of 3D M@MO composites on a one-dimensional fiber, leading to the development of high performance chemical sensors with high specific surface area and excellent gas accessibility. In addition, M@MO composites act as synergistic catalysts due to the heterojunctions between metal oxide frame and the SMO NFs. Preparative process diagram of the Pd@ZnO-WO_3_ NFs is shown in the Figure 6a. TEM images of the samples are shown in the Figure 6b, and inset is its high magnification TEM image. Pd@ZIF-8 particles are embedded on the surface of ammonium metatungstate hydrate (AMH)/PVP NFs and composite NFs. Dual heterogeneous multi-junctions appears on the interface between Pd-ZnO and ZnO-WO_3_ via electrospinning, which promotes electron conduction and sensing performance of Pd@ZnO-WO_3_ NFs. Kim and co-workers have conducted further research on the basis of this work and prepared the PdO@ZnO dual catalysts-loaded hollow SnO_2_ NTs (PdO@ZnO-SnO_2_ NTs) via electrospinning and the subsequent calcination [61]. The hollow structure in PdO@ZnO-SnO_2_ NTs can ensure the uniform loading of a large amount of precious metal particles, and the active sites are also increased correspondingly. The hollow PdO@ZnO-SnO_2_ NTs exhibited high acetone response (Rair/Rgas = 5.06 at 400 °C@1 ppm). Guo et al. have prepared the Pt-ZnO-In_2_O_3_ composite nanofibers with ZIF-8 as a template by electrospinning [91]. The excellent acetone gas sensing property of Pt-ZnO-In_2_O_3_ NFs is related to the n-n heterojunctions between ZnO and In_2_O_3_ and p-n heterojunction formed between p-type PtO_2_ and n-type In_2_O_3_ (ZnO). Precious metal particles can be uniformly dispersed in the network structure formed by electrospun nanofibers and MOF frames, which can effectively solve the deterioration of sensing performance caused by agglomeration of metal particles. After heat treatment, a variety of heterojunctions formed in the material can enhance the conductivity and the synergy among the metal oxides, and a large number of oxygen vacancies can improve the sensing performance.

#### 3.2.4. Electrocatalyst

Fuel cells are receiving more attentions with the development of clean energy technologies [92,93]. In general, oxygen reduction reaction (ORR) plays a key role in fuel cells, metal-air batteries, and water decomposition [94]. Pt and its alloys are considered to be the most effective ORR electrocatalyst at present, but their high cost and poor stability stimulate the increasing interests to develop new materials as potential replacements [95]. However, the materials currently developed still possesses the disadvantages such as low conductivity, environmental pollution, and unsatisfactory performance. Electrospinning is an important method for preparing carbon nanofibers with large specific surface area, good electrical conductivity, excellent mechanical strength, and good chemical stability [96]. MOFs are precursors and templates that are well suited for the preparation of porous carbon materials. Thus MOF-derived carbon-based materials have attracted increasing attention in ORR catalysis due to their adjustable structure, and porous characteristics. Due to the importance of the inherent transition metals or heteroatoms, the performance of electrospun MOF-derived electrocatalysts can be optimized by selecting suitable metal (or metal cluster) centers and organic ligands. For instance, Niu et al. have designed Zn-Co bimetallic nitrogen doped hollow carbon nanofiber composite for ORR application [97]. 

**Figure 7:**
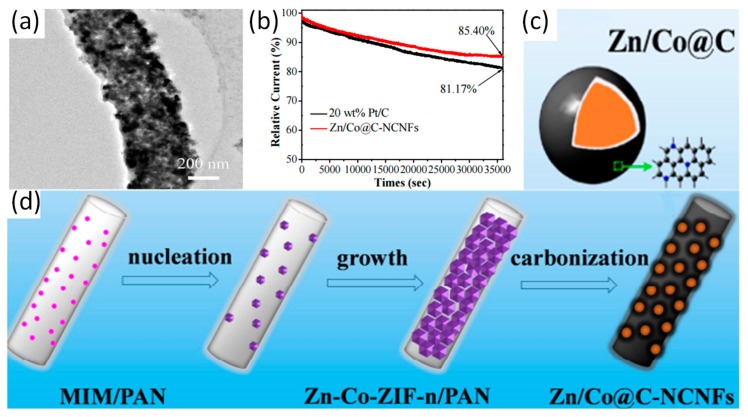
(**a**) TEM image of the Zn/Co@C-NCNFs; (**b**) Durability evaluation from current versus time chronoamperometric responses of the Zn/Co@C-NCNFs and 20 wt % Pt/C electrodes; Schematic illustration of (**c**) Core–shell structure and (**d**) the preparation of Zn/Co@C-NCNFs. Reproduced from [97], with permission from Elsevier, 2016.

Bimetall zeolitic imidazolate frameworks (ZIFs) grown on the surface of MIM/PAN electrospun nanofibers via electrospinning, that is Zn-Co-ZIF-n(shell)/PAN(core) nanofibers (n refers to the molar ratio of Zn/Co before carbonization). After subsequent calcination, Zn/Co@C-NCNFs with layered network structure and high surface area are prepared, whose core layer is nitrogen-doped carbon nanofibers (NCNFs) and the shell layer is Zn/Co bimetallic nanoparticles coated with graphitic carbon layer (Zn/Co@C). Preparative process of Zn/Co@C-NCNFs is shown in Figure 7d. The diameter of the nanofibers gradually increases, which is corresponding to the growth process of nanofibers. The results illustrate that metal ions and organic ligands nucleate by coordination and grow on nanofibers. As the particles are limited by the surface of the fiber during growth, the smaller particle can be obtained easily. Moreover, Zn/Co@C-NCNFs displays core–shell nanofiber structure with dual active centers, and the formation of the graphitic layer in Zn/Co@C-NCNFs are beneficial to the conduction of electrons. The result showed that the duration stability of the Zn/Co@C-NCNFs was far better than that of the commercially available 20 wt % Pt/C (Figure 7b). Graphite carbon formed by carbonization can significantly improve the conductivity of materials. In addition, doped N combines with graphite carbon under high-temperature carbonization, and the formation of mesoporous structure can enhance the electrochemical properties of the material. Guo et al. have reported a novel pod-like nitrogen-doped carbon fibers encapsulated with cobalt/cobalt oxide nanoparticles (Co/CoO_X_-N-C), which were prepared by electrospinning and carbonization with ZIF-67 used as a template [62]. The Co/CoO_X_-N-C exhibited excellent discharge specific capacity (610 mAh g^−1^) and discharge voltage, which are comparable to 20 wt % Pt/C and RuO_2_. Moreover, Wu et al. have reported that hierarchical porous Fe-N-doped carbon nanofibers (Fe-NHCFs) can be fabricated via carbonization of MOF nanofibers [98]. Fe-N doping induces a large number of active sites to enhance the electrocatalytic activity of materials. Parallel characteristics with Pt/C is obtained in an assembled Zn-air battery, which provides a discharge current density of 90 mA cm^−2^ and an output peak power density of 61 mW cm^−2^. Application performances or properties of representative electrospun MOF-derived carbon nanofibers are shown in Table 3.

MOF-derived carbon-based materials doped with heteroatom can exhibit electrocatalytic properties comparable to those of Pt, which mainly due to the following aspects:

(a) The inherent MOF framework and pore canals formed by interweaving the electrospun fibers with the organic framework during the carbonization process provide additional channels for O_2_ in the system (promoting mass transfer); 

(b) Highly graphitized and one-dimensional porous conductive networks improve the conductivity and electron transfer of materials; 

(c) Metal-heteroatom doping in the system induces the formation of a large number of active sites.

## 4. Summary and Outlook

Due to the existence of heterojunction between MOF nanoparticles and nanofibers, the stability of the materials are enhanced while active sites are increased in number. In addition, the anxiety regarding the difficulty of recycling and easy agglomeration of the nanoparticle can be relieved with the adoption of the electrospun fibers support. After calcination, the mesoporous structure, defects and oxygen vacancies may be formed in the electrospun MOF-derived nanomaterials (mainly referring to the materials after heat treatment), which is beneficial for the improvement of the performance of the obtained composite nanofibers. However, further efforts in the field are still needed to address the following issues: 

(1) For M-NFs composite materials: (a) the activity and specific surface area of the material decrease compared to their constituent MOFs nanoparticles; (b) The loading of MOFs particles in the electrospinning method is less controllable and the stable growth of MOFs particles on the fibers is hard to be achieved during in situ growth process. 

(2) For the electrospun MOF-derived nanomaterials, the mechanical properties of the fibers after heat treatment are significantly reduced, hindering its further application. 

(3) More research should focus on the relationship between performance and structure. In other words, M-NFs and their derivatives obtained by electrospinning have great potential in many emerging applications.

## Figures and Tables

**Table 1:** BET surface area of ZIF-8/polymers nanofibers.

Sample	ZIF-8 Concentration (wt %)	BET surface area/m^2^ g^−1^	Ref.
ZIF-8	100	960	[33]
ZIF-8/PVP	22	180	[33]
ZIF-8/PVP	56	530	[33]
PVP	0	10	[33]
ZIF-8/PS	25	210	[33]
ZIF-8	100	1195	[34]
ZIF-8/PU	63	566	[34]
ZIF-8	100	1219	[34]
PAN@ZIF-8	-	983	[35]

**Table 2:** Application performances or properties of representative M-NFs.

M-NFs	Metal center of MOFs	Property or application	Value	Ref.
ZIF-8/PVDF	Zn	Oil/water separation	Rejection rate 92.93%	[29]
Co-MOF/PLA	Co	Antimicrobial mats	-	[31]
ZIF-67/PLA	Co	Antibacterial film	-	[32]
ZIF-8/PU	Zn	BET surface area	566 cm^2^ g ^−1^	[34]
ZIF-8/PAN	Zn	BET surface area	983 cm^2^ g ^−1^	[35]
HKUST-1/PAN	Cu	CO_2_ capture	3.9 mmol g^−1^	[40]
UiO-66-NH_2_/PS	Zr	Chemical warfare agent removal	Soman half-lives (t_1/2_) 95 min	[43]
MOF-808/PAN	Zr	Heavy metal ions removal	Adsorption capacities (225.05 mg g^−1^ for Cd^2+^ 287.06 mg g^−1^ for Zn^2+^)	[47]
HKUST-1/Cellulose	Cu	BET surface area	Increased 44 to 440 m^2^ g^−1^	[52]
MOF-5/Cellulose	Zn	Gas adsorption.	-	[53]
Zr-MOF/PA-6	Zr	Degradation of CWAs	Half-lives of nerve agent soman 2.3 min	[54]
Zn-MOF/SPPESK	Zn	Proton exchange membrane fuel cell	Proton conductivity (8.2 ± 0.16) × 10^−2^ S cm^−1^ (160 °C)	[55]
ZIF-8/CS-PEO	Zn	Antimicrobial mats	100% antibacterial activity against Gram-positive *Staphylococcus aureus*	[56]
MOF-199/PS	Cu	Determination of acetaldehyde in human urine	Limits of detection 0.01 to 0.02 ng mL^−1^	[57]
UiO-66/PAN	Zr	Determination of plant hormone content	Limit of detection 0.01 ng mL^−1^	[58]
MOF-5/PAN	Zn	Solid-phase extraction of two estrogenic drugs in urine samples	Limit of detection 0.02 g L^−1^	[59]
ZIF-8/PAN	Zn	Functional textiles with filtration function	Removal of PM2.5 78.35%	[60]

**Table 3:** Application performances or properties of conventional carbon nanocomposites and representative electrospun MOFs-derived carbon nanofibers.

Composites		Precursor (or Template)	Property (or Application)	Value	Ref.
Mn_3_O_4_/graphene		Mn/GO	Lithium ion battery	Reversible capacity of 500 mAh g^−1^ at a current density of 60 mA g^−1^	[67]
Mn_3_O_4_@C		Mn/PVP	Lithium ion battery	Reversible capacity of 473 mAh g^−1^ at a current density of 40 mA g^−1^	[68]
Co_3_O_4_/C		ZIF-67/PAN	Lithium ion battery	Capacity of 1024.1mAh g^−1^ after 100 cycles	[69]
SnO_2_-Co_3_O_4_ NFs		ZIF-67	Lithium ion battery	Reversible capacity of 1287 mAh g^–1^ after 300 cycles	[70]
MnO_x_/CNFs		Mn-MOF	Lithium ion battery	Prolonged stability over 1000 cycles	[72]
NiCo_2_O_4_/NiO/CNFs		Ni/Co-MOF	Sodium ion battery	Sodium-storage capacity of 210 mAh g^−1^	[73]
Co_3_O_4_@CNFs		ZIF-67/PAN	Lithium ion battery	The reversible capacity 558 mAh g^−1^ after 500 cycles at 5 A g^−1^	[74]
Mn_3_O_4_/NPCs		Mn-MOF	Lithium ion battery	Specific capacity (1058 mAh g^−1^ at 50 mA g^−1^)	[99]
N-doped Co/CoO_x_ CNFs		ZIF-67@PAN	Zn-air battery	Discharge specific capacity of 610 mAh g^−1^	[62]
Fe-N-doped CNFs		Zn-Fe-ZIF/PAN	Zn-air battery	Comparable with the commercial 20wt % Pt/C	[98]
Exfoliated-CNTs		Multi-walled carbon nanotubes	Supercapacitors	Specific capacitance of 165 F g^−1^ at current density of 5 A g^−1^	[77]
Graphene aerogels(GAs)		GO	Supercapacitors	-	[78]
Graphitized polyimide web		Pyromellitic dianhydride (PMDA)/4,4’-oxydianiline (ODA)	Supercapacitors	Specific capacitance 175 F g^−1^ at current density of 1000 mA g^−1^	[82]
N-doped graphitic hierarchically porous carbon nanofibers (NGHPCF)		Zn/Co-MOF	Supercapacitors	Specific capacitance of 326 F g^−1^ at current density of 0.5 A g^−1^	[85]
Pd@MOF-5		MOF-5	-	-	[87]
Au@Ag/ZIF-8		ZIF-8	Catalysis	-	[88]
PdO@ZnO-SnO_2_ NTs	Pd@ZIF-8	Acetone sensor	Rair/Rgas = 5.06 at 400 °C@1 ppm		[61]
Pt@ZnOTiO_2_ NTs	Pt@ZIF-8	Toluene sensor	Detection limit (23 parts per billion)		[100]
Pt-ZnO-In_2_O_3_ NFs	Pt@ZIF-8	Acetone sensor	Response and recovery times to 100 ppm acetone (1/44 s) at 300 °C		[91]
Zn/Co@C-NCNFs		Zn-Co-ZIF/PAN	ORR	Electron selectivity 3.69	[97]
Pt@MIL-101@PCL		MIL-101	Hydrogenation catalyst	Complete reaction within 90 min	[45]
